# Supplementary material for: Diverse Mechanisms of Resistance against Osimertinib, a Third-Generation EGFR-TKI, in Lung Adenocarcinoma Cells with an EGFR-Activating Mutation
Source: Cells. 2022 Jul 14;11(14):2201. doi: 10.3390/cells11142201 (PMC9319811; doi:10.3390/cells11142201)
Supplement: Supplementary file 1 [file cells-11-02201-s001.zip › cells-1744166-supplementary.pdf]

Table S1

The primary antibodies used in this study

Antibodies	Source	Identifier
anti-total EGFR	Cell Signaling Technology	#4267
anti-phospho-EGFR (Y1068)	Cell Signaling Technology	#3777
anti-EGFR (E746-A750del)	Cell Signaling Technology	#2085
anti-total HER2	Cell Signaling Technology	#4290
anti-total insulin-like growth factor 1 receptor (IGF1R)	Cell Signaling Technology	#3018
anti-phospho-IGF1R	Cell Signaling Technology	#3024
anti-total MET	Cell Signaling Technology	#8198
anti-total AKT	Cell Signaling Technology	#9272
anti-phospho-AKT (S473)	Cell Signaling Technology	#9271
anti-total extracellular signal-regulated kinase (ERK1/2)	Cell Signaling Technology	#9102
anti-phospho-ERK1/2	Cell Signaling Technology	#4370
anti-total MAPK (MEK1/2)	Cell Signaling Technology	#9126
anti-phospho-MAPK kinase (MEK1/2)	Cell Signaling Technology	#2338
anti-cleaved PARP	Cell Signaling Technology	#5625
anti-Bcl-2	Cell Signaling Technology	#2870
anti-Bcl-xL	Cell Signaling Technology	#2764
anti-Mcl-1	Cell Signaling Technology	#94296
anti-phospho-Bad (S136)	Cell Signaling Technology	#4366
anti-total Bad	Cell Signaling Technology	#9236
anti-Bim	Cell Signaling Technology	#2933
anti-Bid	Cell Signaling Technology	#2002
anti-Puma	Cell Signaling Technology	#4976
anti-Bax	Cell Signaling Technology	#5023
anti-Bak	Cell Signaling Technology	#6947
anti-cleaved caspase-3	Cell Signaling Technology	#9664
anti-total caspase-3	Cell Signaling Technology	#9665
anti-caspase-8	Cell Signaling Technology	#9746
anti-cytochrome c	Cell Signaling Technology	#11940
anti-phospho Gab1	Cell Signaling Technology	#3233
anti-total Gab1	Cell Signaling Technology	#3232
anti-phospho SHP2	Cell Signaling Technology	#3751
anti-total SHP2	Cell Signaling Technology	#3397
anti-β-actin	Cell Signaling Technology	#4970
KRAS	Santa Cruz Biotechnology	sc-30
HRAS	Santa Cruz Biotechnology	sc-29
NRAS	Santa Cruz Biotechnology	sc-31
Anti-Bax monoclonal antibody clone 6A7	EMD Millipore	MABC1176

Table S2

RT-PCR primers (gDNA)

Genes		Primer sequences (5' to 3')
EGFR	F	CAAGGCCATGGAATCTGTCA
	R	CTGGAATGAGGTGGAGGAACA
KRAS	F	CACCCTAGACAAGCAGCCAATA
	R	AAGCCCTGCCGCAAAAA
Line-1	F	AAAGCCGCTCAACTACATGG
	R	TGCTTTGAATGCGTCCCAGAG

Sequence primers

Genes		Primer sequences (5' to 3')
EGFR exon 19	F	GCAATATCAGCCTTAGGTGCGGCTC
	R	CATAGAAAGTGAACATTTAGGATGTG
KRAS exon 2	F	GTATTAACCTTATGTGTGACA
	R	GTCCTGCACCAGTAATATGC
PTPN11 exon 13	F	GTCTCTGAGTCCACTAAAAGTTGTGC
	R	AGCGTATCCAAGAGGCCTAGC

Figure S1

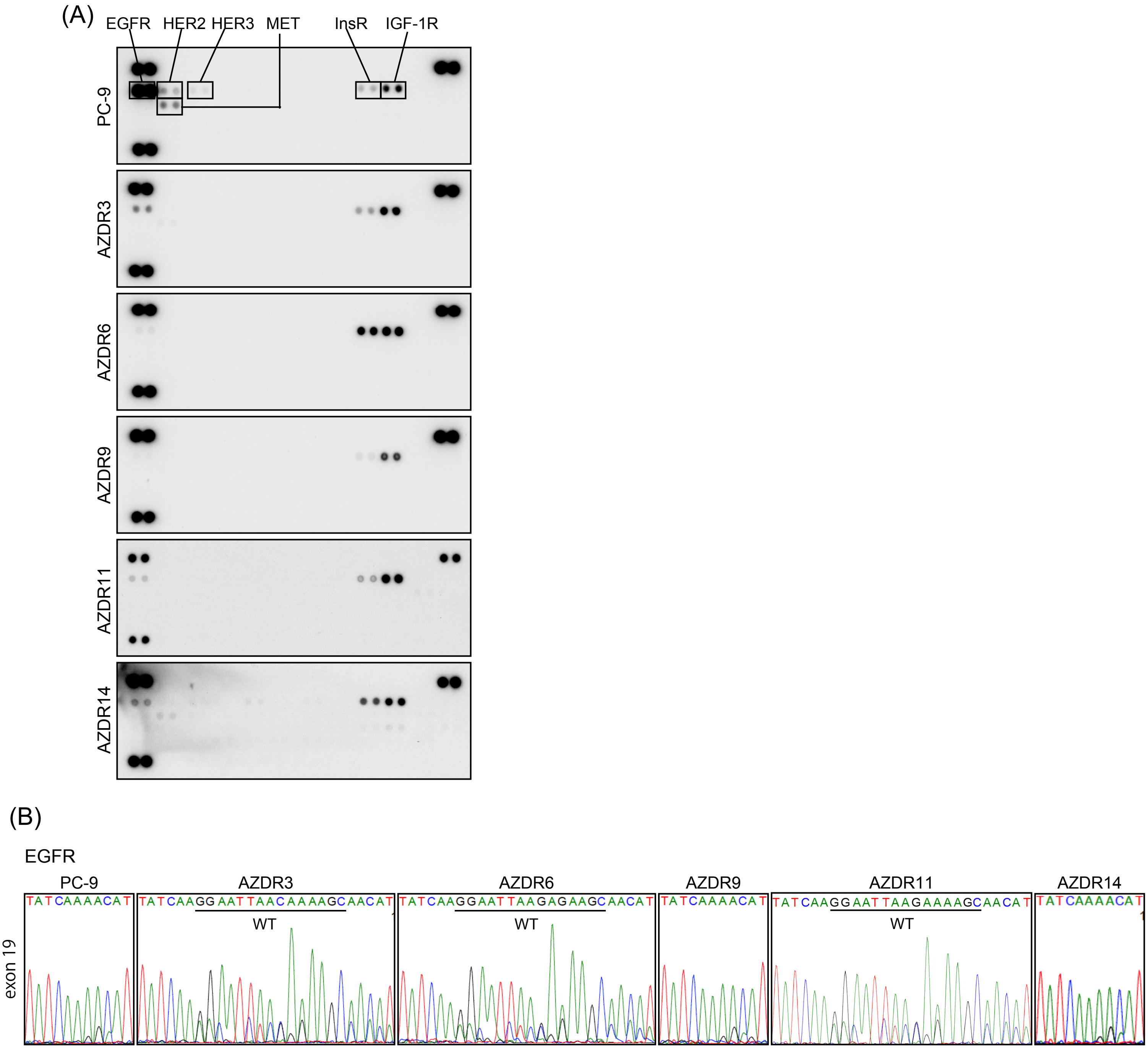
(A)
EGFR
HER2
HER3
MET
InsR
IGF-1R
PC-9
AZDR3
AZDR6
AZDR9
AZDR11
AZDR14
(B)
EGFR
PC-9
AZDR3
AZDR6
AZDR9
AZDR11
AZDR14
exon 19
WT
WT
WT

Figure S2

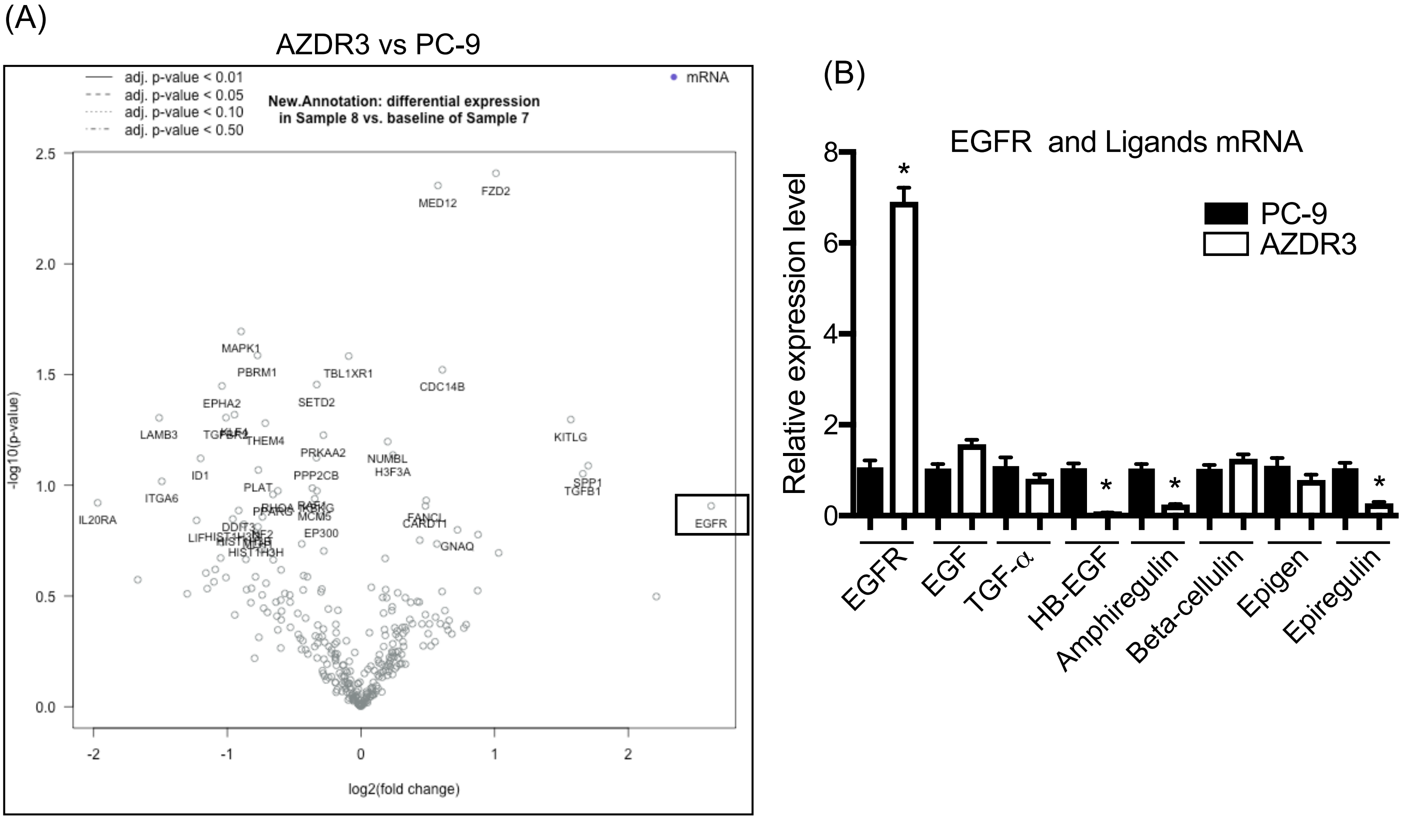
(A)
AZDR3 vs PC-9
adj. p-value < 0.01
adj. p-value < 0.05
adj. p-value < 0.10
adj. p-value < 0.50
New.Annotation: differential expression
in Sample 8 vs. baseline of Sample 7
mRNA
-log10(p-value)
log2(fold change)
MED12
FZD2
MAPK1
PBRM1
TBL1XR1
CDC14B
EPHA2
SETD2
LAMB3
THEM4
KITLG
PRKAA2
NUMBL
H3F3A
ID1
PPP2CB
SPP1
TGFB1
ITGA6
PLAT
IL20RA
EGFR
EP300
GNAQ
HIST1H3H
(B)
EGFR and Ligands mRNA
PC-9
AZDR3
Relative expression level
EGFR
EGF
TGF-α
HB-EGF
Amphiregulin
Beta-cellulin
Epigen
Epiregulin

Figure S3

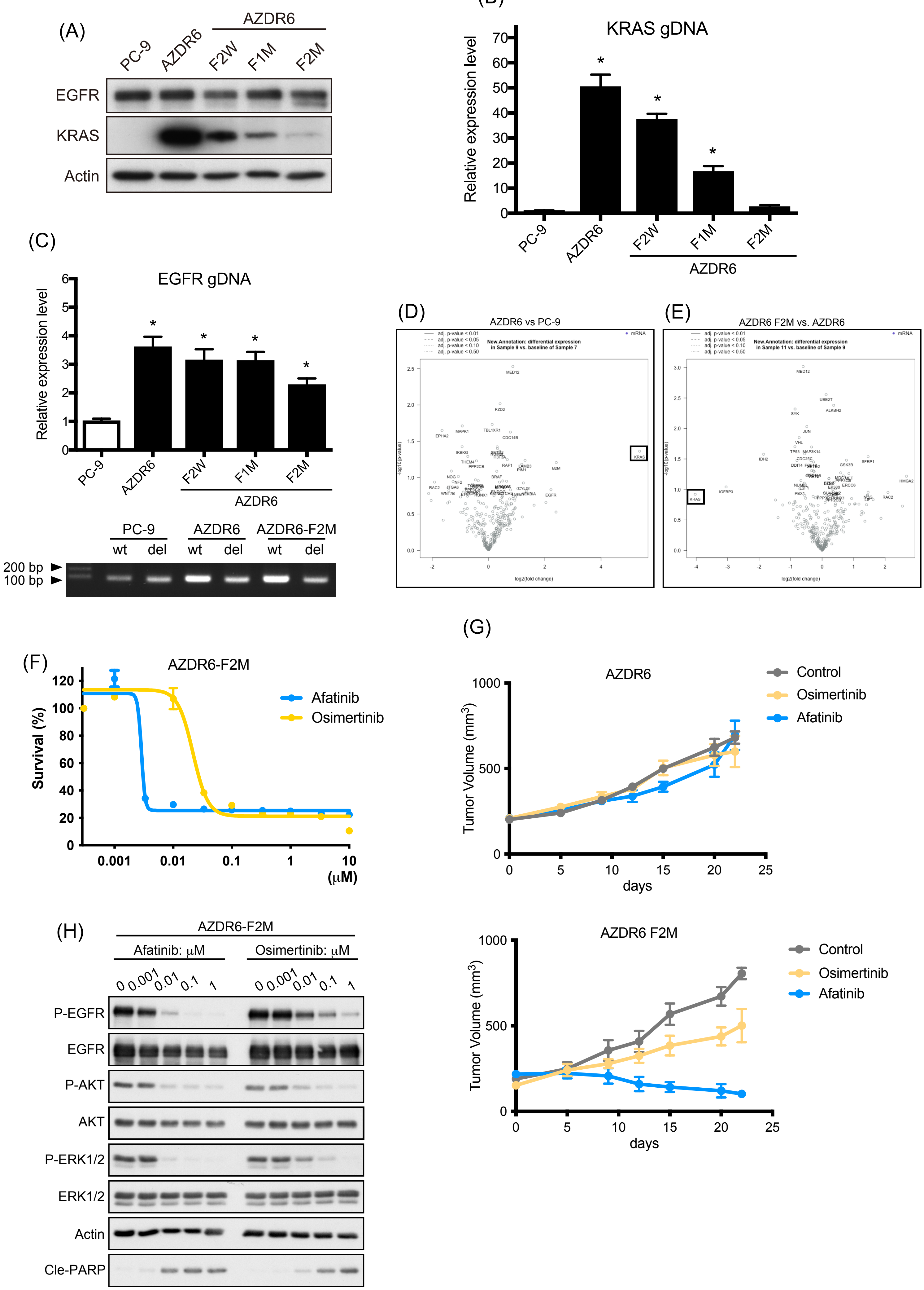
(A)
AZDR6
PC-9
AZDR6
F2W
F1M
F2M
EGFR
KRAS
Actin
(B)
KRAS gDNA
Relative expression level
0
10
20
30
40
50
60
70
*
*
*
PC-9
AZDR6
F2W
F1M
F2M
AZDR6
(C)
EGFR gDNA
Relative expression level
0
1
2
3
4
5
6
*
*
*
*
PC-9
AZDR6
F2W
F1M
F2M
AZDR6
PC-9
AZDR6
AZDR6-F2M
wt
del
wt
del
wt
del
200 bp
100 bp
(D)
AZDR6 vs PC-9
KRAS
MED12
EGFR
log2(fold change)
(E)
AZDR6 F2M vs. AZDR6
KRAS
MED12
log2(fold change)
(F)
AZDR6-F2M
Afatinib
Osimertinib
Survival (%)
0
20
40
60
80
100
120
0.001
0.01
0.1
1
10
(μM)
(G)
AZDR6
Control
Osimertinib
Afatinib
Tumor Volume (mm3)
0
500
1000
0
5
10
15
20
25
days
AZDR6 F2M
Control
Osimertinib
Afatinib
Tumor Volume (mm3)
0
500
1000
0
5
10
15
20
25
days
(H)
AZDR6-F2M
Afatinib: μM
Osimertinib: μM
0
0.001
0.01
0.1
1
0
0.001
0.01
0.1
1
P-EGFR
EGFR
P-AKT
AKT
P-ERK1/2
ERK1/2
Actin
Cle-PARP

Figure S4

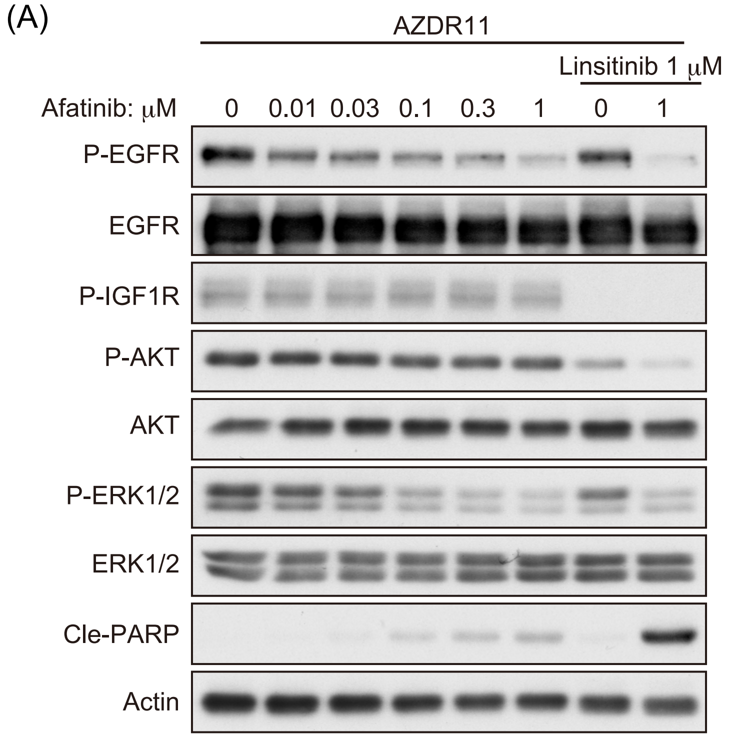
(A)
AZDR11
Linsitinib 1 μM
Afatinib: μM
0
0.01
0.03
0.1
0.3
1
0
1
P-EGFR
EGFR
P-IGF1R
P-AKT
AKT
P-ERK1/2
ERK1/2
Cle-PARP
Actin

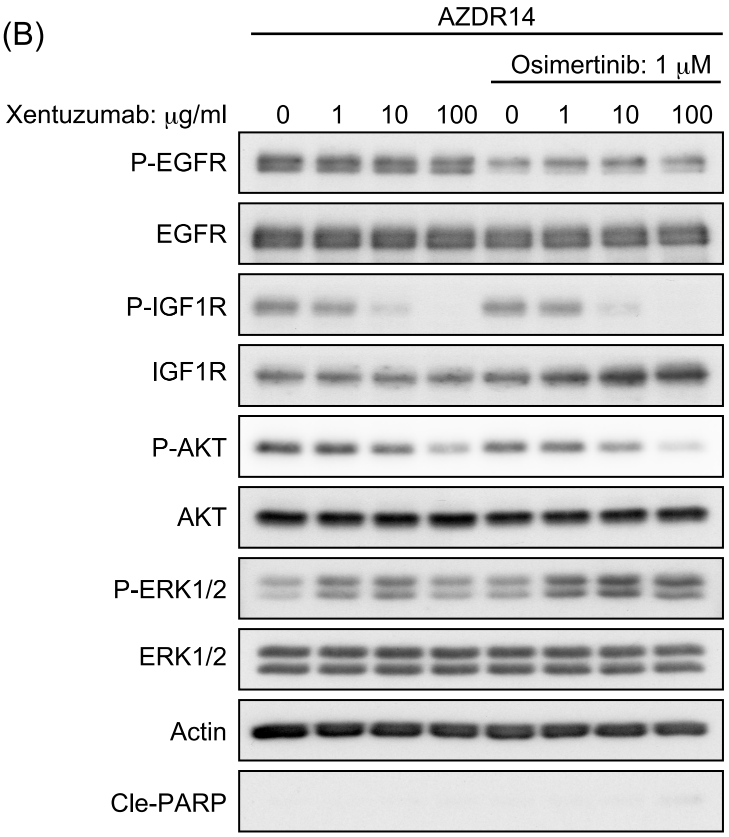
(B)
AZDR14
Osimertinib: 1 μM
Xentuzumab: μg/ml
0
1
10
100
0
1
10
100
P-EGFR
EGFR
P-IGF1R
IGF1R
P-AKT
AKT
P-ERK1/2
ERK1/2
Actin
Cle-PARP
